# The prognostic value of ^123^I-mIBG SPECT cardiac imaging in heart failure patients: a systematic review

**DOI:** 10.1007/s12350-020-02501-w

**Published:** 2021-01-13

**Authors:** Mariano Pontico, Gabriele Brunotti, Miriam Conte, Ferdinando Corica, Laura Cosma, Cristina De Angelis, Maria Silvia De Feo, Julia Lazri, Antonio Matto, Melissa Montebello, Arianna Di Rocco, Viviana Frantellizzi, Alessio Farcomeni, Giuseppe De Vincentis

**Affiliations:** 1 Department of Radiological Sciences, Oncology and Anatomical Pathology, Sapienza University of Rome, Viale Regina Elena 324, 00161 Rome, Italy; 2 Department of Public Health and Infectious Diseases, “Sapienza” University of Rome, Rome, Italy; 3 Department of Economics & Finance, University of Rome “Tor Vergata”, Rome, Italy

**Keywords:** ^123^I-mIBG, SPECT, heart failure, arrhythmic event, sudden cardiac death

## Abstract

This systematic review aimed to evaluate the prognostic value of Iodine123 Metaiodobenzylguanidine (^123^I-mIBG) SPECT myocardial imaging in patients with heart failure (HF) and to assess whether semi-quantitative SPECT scores can be useful for accurate risk stratification concerning arrhythmic event (AE) and sudden cardiac death (SCD) in this cohort. A systematic literature search of studies published until November 2020 regarding the application of ^123^I-mIBG SPECT in HF patients was performed, in Pubmed, Scopus, Medline, Central (Cochrane Library) and Web Of Science databases, including the words “MIBG”, “metaiodobenzylguanidine”, “heart”, “spect”, and “tomographic”. The included studies had to correlate ^123^I-mIBG SPECT scores with endpoints such as overall survival and prevention of AE and SCD in HF patients. According to the sixteen studies included, the analysis showed that 123I-mIBG SPECT scores, such as summed defect score (SDS), regional wash-out (rWO), and regional myocardial tracer uptake, could have a reliable prognostic value in patients with HF. An increased SDS or rWO, as well as a reduced ^123^I-mIBG myocardial uptake, have proven to be effective in predicting AE- and SCD-specific risk in HF patients. Despite achieved results being promising, a more reproducible standardized method for semi-quantitative analysis and further studies with larger cohort are needed for ^123^I-mIBG SPECT myocardial imaging to be as reliable and, thus, accepted as the conventional ^123^I-mIBG planar myocardial imaging.

**Supplementary information:**

The online version of this article (10.1007/s12350-020-02501-w) contains supplementary material, which is available to authorized users.

## Introduction

Heart failure (HF) is characterized by several abnormalities of sympathetic cardiac activity: an increased sympathetic response is initially favorable by serving as compensation for decreased cardiac output, but as HF progresses this response leads to deleterious neurohormonal and myocardial structural changes that worsen the condition and increase the likelihood of adverse cardiac events.^1^

Iodine123-Metaiodobenzylguanidine (^123^I-mIBG), an analog of norepinephrine, is a useful tool for detecting abnormalities in the myocardial adrenergic nervous system in HF patients and can be successfully used to assess their prognosis.^2^^–^4^123^I-mIBG represents a tracer of sympathetic neuron integrity and function and the most widely used imaging agent for studying the causes and effects of cardiac sympathetic hyperactivity.^5^ Semi-quantitative myocardial ^123^I-mIBG parameters have proved to be of prognostic value in HF.^6^,^7^ Specifically, some trials were designed to assess the capability of the heart to mediastinum ratio (H/M) on ^123^I-mIBG planar scintigraphy for predicting prognosis for significant adverse cardiac events in subjects with HF.^8^ Currently, the H/M is the basis for the clinical decision-making diagnostic work-up, enabling the common application of a single standardized parameter beyond worldwide institutions.^9^,^10^ Several single and multicenter studies demonstrated and confirmed its potent prognostic role in the clinical evaluation and risk stratification in HF.^11^,^12^ HF patients with impaired myocardial ^123^I-mIBG parameters had a worse prognosis compared with those with relatively preserved parameters (i.e., reduced late H/M and increased myocardial wash-out rate (WR)).^13^ However, it has become progressively more common to include single-photon emission computerized tomography (SPECT) imaging in clinical and research protocol.^14^ Myocardial SPECT imaging allows evaluation of the regional sympathetic activity and polar maps of the myocardium could be obtained by SPECT images, providing an assessment of the defect extent and severity.^15^,^16^ Furthermore, the ability of ^123^I-mIBG SPECT to provide regional information not available on planar images remains a driver for efforts to incorporate this procedure into assessments of HF patients with arrhythmic events (AE) and sudden cardiac death (SCD) specific risk.^17^,^18^ Despite PET imaging holds higher spatial resolution and superior quantitative capabilities,^19^,^20^ for the forthcoming future ^123^I-mIBG SPECT accounts for the most widely available nuclear imaging technique for an accurate assessment of the regional myocardial sympathetic innervation.^21^ Nonetheless, a shared and established consent of these ^123^I-mIBG SPECT parameters is found to be hard to get according to the present knowledge. Therefore, to date, there is an urgent need for improved risk stratification for patients developing HF and growing evidence that ^123^I-mIBG SPECT imaging should gain greater clinical relevance. The aim of this systematic review is to evaluate the prognostic value of ^123^I-mIBG SPECT myocardial imaging in patients with HF and to assess whether semi-quantitative SPECT scores can be useful for accurate risk stratification with regard to AE and SCD.

## Materials and Methods

### Search Strategy and Study Selection

This systematic review was drawn up following PRISMA guidelines.^22^ An online literature search looking up articles that suited the inclusion criteria was conducted on Pubmed, Scopus, Medline, Central (Cochrane Library), and Web Of Science databases. Papers published from January 1950 to November 2020 concerning HF patients assessed by means of ^123^I-mIBG SPECT were searched. The applied search query was the following: ((MIBG* [WORD] OR metaiodobenzylguanidine [WORD]) AND (heart [WORD]) AND (spect [WORD] OR tomographic [WORD])). This string was then adapted for each database. Eligible studies had to take into consideration semi-quantitative scores expressly derived by myocardial SPECT imaging, like regional wash-out rate (rWO) and summed scores (SS) values. Studies were included when these scores were correlated with specific endpoints, such as overall survival (OS) and prevention of AE and SCD. Reviews, case reports, editorials, studies conducted on animals or phantoms, and studies concerning oncologic conditions were excluded. The English language was mandatory for inclusion in the analysis. References of the provided articles were also examined to find out any additional relevant studies.

### Data Extraction and Methodological Quality Assessment

For each included study, general data about the article (authors, journal, year of publication, country, and study design) along with patient characteristics were retrieved.

The methodological quality of included studies was assessed using the Quality Assessment of Diagnostic accuracy Studies-2 (QUADAS2) tool. Since its publication in 2003,^23^ the QUADAS tool has been widely used^24^,^25^ and it is recommended for use in systematic reviews of diagnostic accuracy by the Agency for Healthcare Research and Quality, Cochrane Collaboration (Cochrane Handbook for Systematic Reviews of Diagnostic Test Accuracy), and the U.K. National Institute for Health and Clinical Excellence. Data extraction and quality assessment were independently performed by two reviewers and eventual disagreements were resolved by unanimous approval after discussion.

## Results

### Search Results

**Figure 1:**
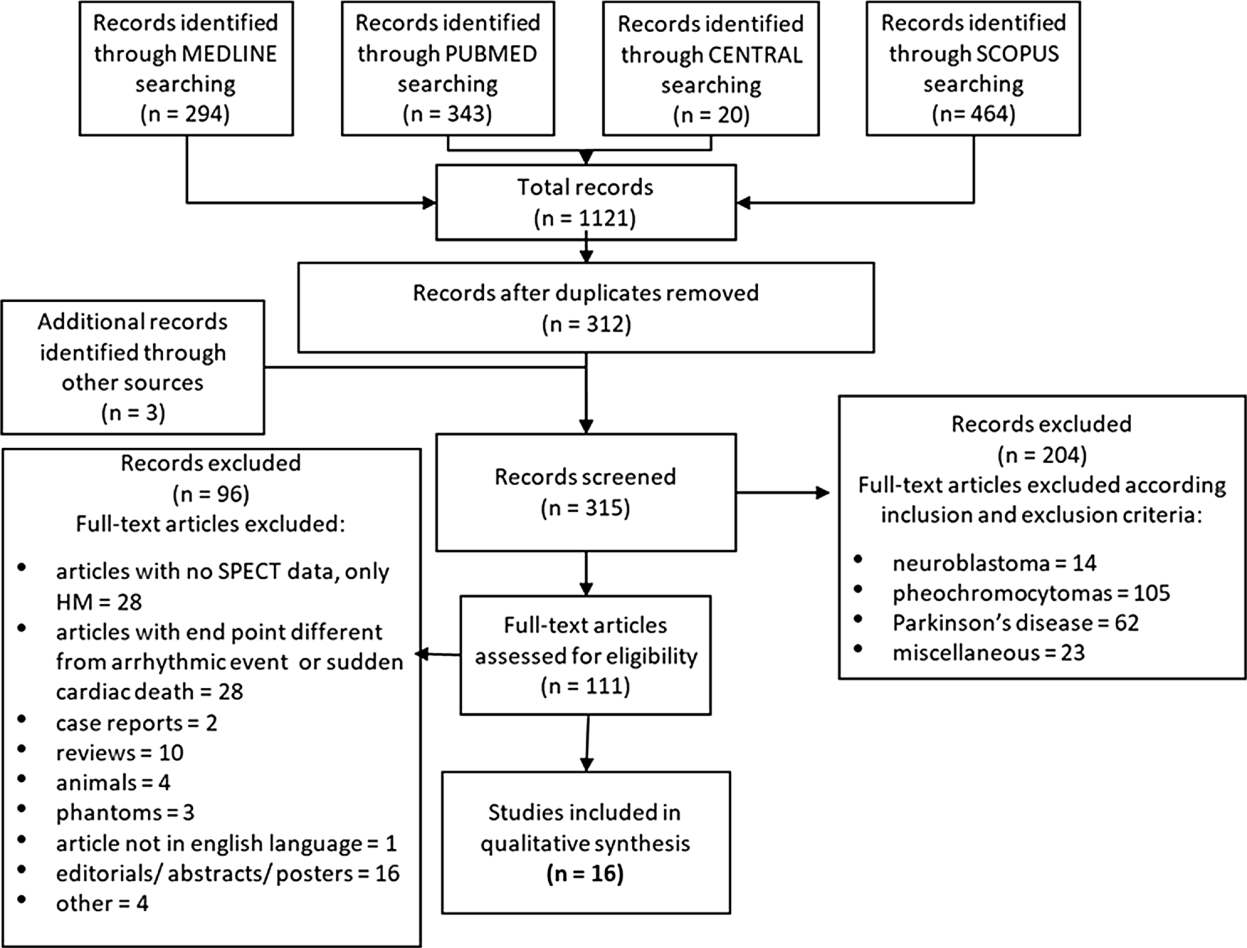
Study selection flow chart

A total of 315 articles were found and thus the authors examined each abstract to identify potentially suitable studies. From the overall group of 315, 204 full-text articles concerning patients with neuroblastoma,^14^ pheochromocytoma (105), and Parkinson’s disease (62) were excluded. 23 articles were excluded because they did not satisfy the inclusion criteria. The 111 remaining papers were assessed for eligibility with the exclusion of 28 papers with no SPECT data. 16 articles were considered suitable and ultimately selected for this review. The detailed study selection flow chart, along with the search strategy and the selection criteria applied, is represented in Figure 1.

### Study Characteristics

Characteristics of the included studies and technical acquisition parameters used to obtain ^123^I-mIBG myocardial SPECT imaging are reported in Tables 1 and 2. The number of enrolled patients in the analyzed studies ranged from 16 to 985, the latter corresponding with the ADMIRE-HF multicenter trial cohort.^26^ The selected articles were published from 1995 to 2020 by researchers mostly from the USA and Europe, but also one from Japan and another study from Australia. Subjects enrolled were essentially patients with stable NYHA Class II or III HF (ischemic or non-ischemic) at risk for an adverse cardiac event (i.e., AE or SCD), including subjects referred for ICD implantation for primary prevention of SCD.

**Table 1:** ^123^I-mIBG SPECT acquisition parameters of the included studies.

Authors	Acquisition time after injection (Min)			
	Early	Late	Collimator	Matrix
Somsen et al.^44^	/	240	MEHR	64 × 64
Travin et al.^28^	10-15	230	LEHR	ns
Van Der Veen et al.^30^	15	240	LEHR	128 × 128
Yamamoto et al.^39^	20	200	LEHR	128 × 128
Clements et al.^36^	ns	ns	ns	ns
Jacobson et al.^8^	15	230	LEHR	64 × 64
Jacobson et al.^26^	25	240	MEHR	ns
Kasama et al.^38^	15	240	LEHR	128 × 128
Marshall et al.^35^	10-15	180-240	MEHR	64 × 64
Verschure et al.^31^	15	240	LEHR	256 × 256
Hage et al.^49^	15	230	LEHR	128 × 128
Iqbal et al.^12^	15	240	LEHR	256 × 256
Boogers et al.^34^	30	180-240	LEHR	128 × 128
De Vincentis et al.^13^	25	250	LEHR	64 × 64
Sazonova et al.^41^	15	240	LEHR	64 × 64
Doi et al.^40^	15-30	240	LEHR	ns

/ not reported; *ns* not specified; *LEHR* low energy high resolution; *MEHR* medium energy high resolution.

**Table 2:** Characteristics of the included studies and ^123^I-mIBG SPECT results evaluation method.

Authors	Score	Evaluation scale	Polar map (segments)	Population
Boogers et. al. 34	ESS 21.6 ± 10.1	0-4	17	116 CHF
	LSS 26.8±10.0			
De Vincentis et al.^13^	ESS 31.4±11.9	0-4	17	170 CHF
	LSS 36.2±12.3			
	DSS − 4.74±8.6			
Verschure et al.^31^	LSS 39.4 ± 15.5	0-4	17	111 CHF
Iqbal et al.^12^	ESS / LSS / MUP	0-4	20	22 CHF
Jacobson et al.^8^	SS	0-4	17	985 CHF
Jacobson et al.^26^	/	0-4	17	961 CHF
Marshall et al.^35^	SDS 29.5±9.9	/	/	27 CHF
	MS 11.9±8.0			
Somsen et al.^44^	/	/	/	16 CHF
Travin et al.^28^	ESS 41.2±12.4	0-4	17	471 CHF
	PS 19.2±11.3			
	MS 22.5±12.8			
Van der Veen et al.^30^	/	/	/	54 patients 28 CHF
Kasama et al.^38^	LSS	0-4	17	208 CHF
Clements et al.^36^	SDyS	0-4	17	938 HF
Doi et al.^40^	/	/	17	468 HF
Yamamoto et al.^39^	SSS	0-4	17	73 CHF
Hage et al.^49^	/	/	/	917 HF
Sazonova et al.^41^	ESS 21-18	0-4	20	80 CHF
	LSS 24-20			

/ not reported; *CHF* congestive heart failure; *HF* heart failure; *ESS* early summed score; *LSS* late summed score; *DSS* defect summed score; *MUP* myocardial uptake indices; *MS* (perfusion/innervation) mismatch score; *PS* perfusion score; *WR* wash-out rate; *UR* uptake ratio; *SDyS* segment dysinnervation score; *SSS* stress summed score

### Methodological Quality

**Figure 2:**
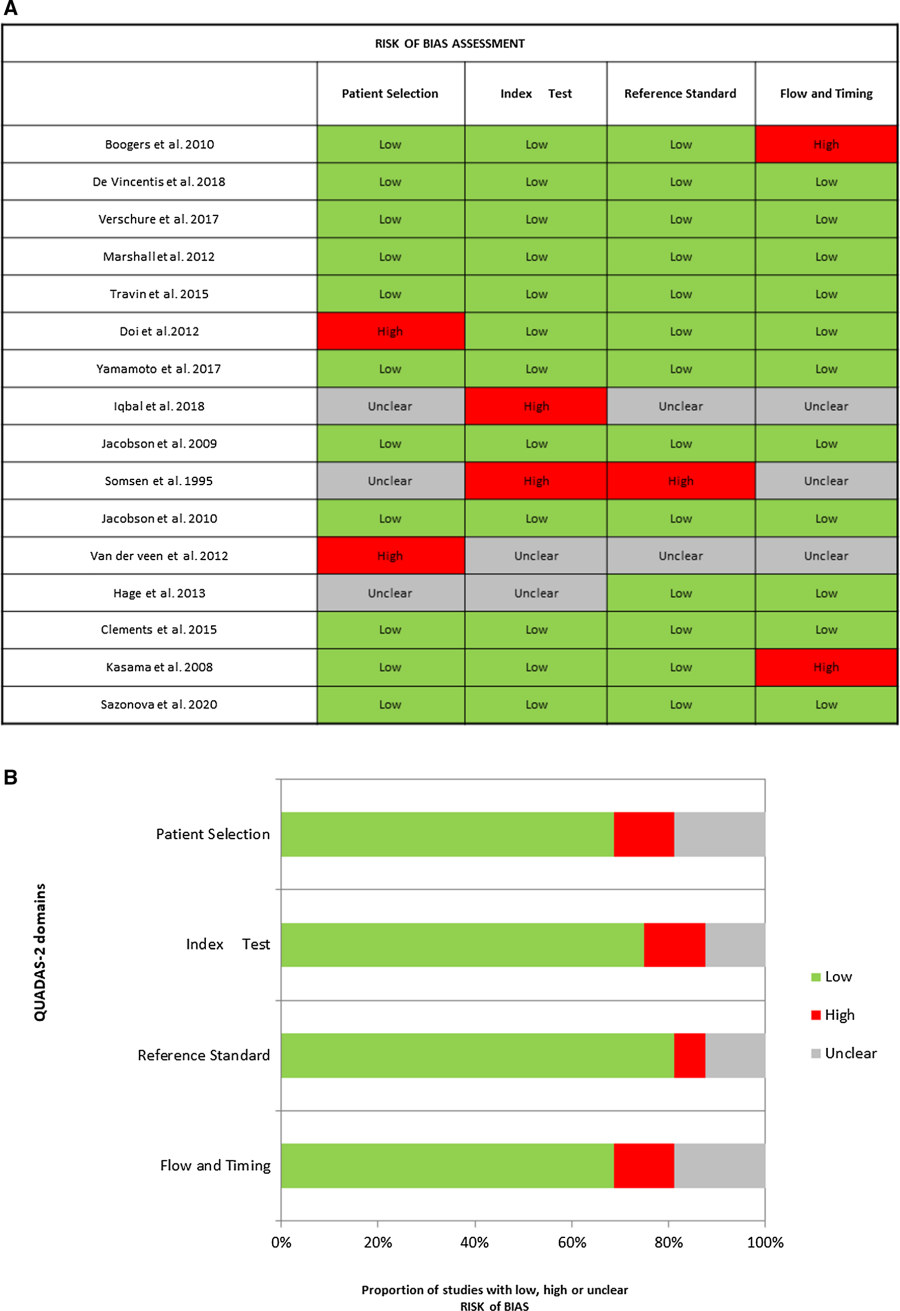
(**A**) Risk of bias assessment through Quadas2 for each study. (**B**) Results of risk of bias assessment through Quadas2 along with its graphic representation

**Figure 3:**
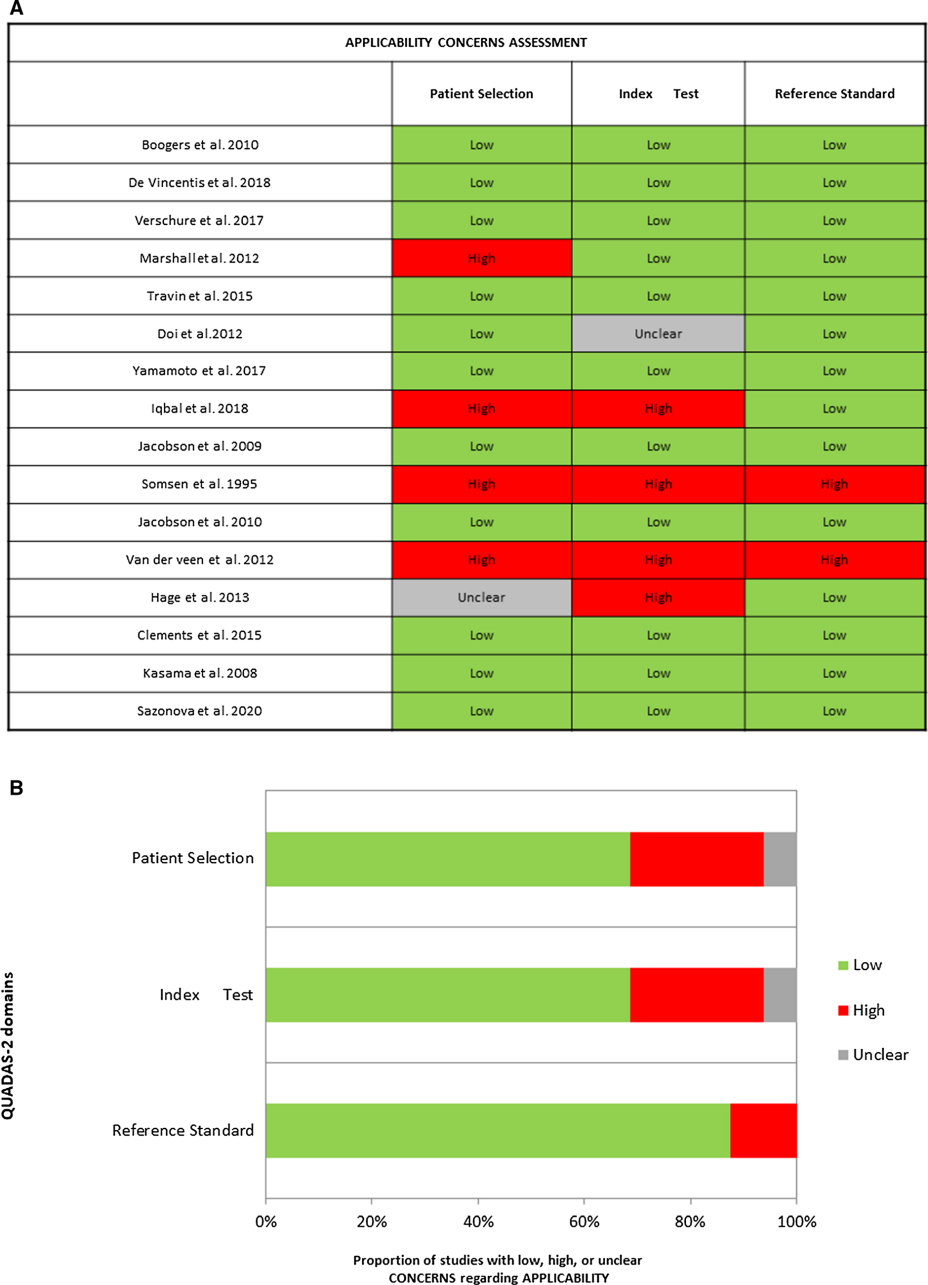
(**A**) Applicability concerns assessment through Quadas2 for each study. (**B**) Results of applicability concerns assessment through Quadas2 along with its graphic representation

The overall methodological quality of the included studies resulted quite good: 12 of 16 studies satisfied at least three and 9 of 16 all of the four QUADAS2 domains for the bias risk assessment and 10 of 16 satisfied each of the three applicability assessment domains, with 12 satisfying at least two domains (Table 3). Considering independently the results within each bias assessment domain, at least 11 studies obtained a low concern of bias and no more than 2 studies showed high risk in some of those (Figure 2A, B). Conversely, taking into account all four bias assessment domains, only two studies reported more than two unclear results, in relation to an insufficient amount of details given to achieve an acceptable methodological protocol appraisal, and only one reported a high risk of bias in two of the four domains. Regarding the patient selection domain, three studies had an unclear risk of bias because there was a lack of detailed data and/or it was not reported whether patients were consecutively enrolled. Two studies reported a high risk of bias because some heterogeneity within the inclusion criteria was detected. Regarding the index test domain, two studies reported a high risk of bias due to some kind of different elaboration and interpretation of the ^123^I-mIBG myocardial SPECT imaging, bringing the risk to decrease the global homogeneity and power of the findings. It was found a high concern of applicability, both in patient selection and index test domains, in three studies. Cumulatively, given four high risks, and one unclear result obtained if considering both patient selection and index test domain, the concern for global applicability was mainly low (Figure 3A, B).

**Table 3:** Tabular representation of quality assessment results

Study	Risk of bias				Applicability concerns		
	Patient selection	Index test	Reference standard	Flow and timing	Patient selection	Index test	Reference standard
Boogers et al.^34^	☺	☺	☺	☹	☺	☺	☺
De Vincentis et al.^13^	☺	☺	☺	☺	☺	☺	☺
Verschure et al.^31^	☺	☺	☺	☺	☺	☺	☺
Marshall et al.^35^	☺	☺	☺	☺	☹	☺	☺
Travin et al.^28^	☺	☺	☺	☺	☺	☺	☺
Doi et al.^40^	☹	☺	☺	☺	☺	?	☺
Yamamoto et al.^39^	☺	☺	☺	☺	☺	☺	☺
Iqbal et al.^12^	?	☹	?	?	☹	☹	☺
Jacobson et al.^8^	☺	☺	☺	☺	☺	☺	☺
Somsen et al.^44^	?	☹	☹	?	☹	☺	☹
Jacobson et al.^26^	☺	☺	☺	☺	☺	☺	☺
Van der veen et al.^30^	☹	?	?	?	☹	☹	☹
Hage et al.^49^	?	?	☺	☺	?	☹	☺
Clements et al.^36^	☺	☺	☺	☺	☺	☺	☺
Kasama et al.^38^	☺	☺	☺	☹	☺	☺	☺
Sazonova et al.^41^	☺	☺	☺	☺	☺	☺	☺

☺Low risk; ☹High risk; ?Unclear risk

### Statistical analysis

A random-effects meta-analysis was also carried out, but it had a very large heterogeneity as measured by the I2. In accordance with the recommendations of the Cochrane Oral Health Group, we therefore decided not to report on this analysis.

## Discussion

In patients with chronic HF, sympathetic hyperactivity initially represents a compensatory mechanism for coping with impaired myocardial function. However, long-term elevated adrenergic stimulation generates a vicious circle which leads in the end to detrimental myocardial remodeling, decline in left ventricular function, and increased morbidity and mortality.^27^ Cardiac sympathetic activity could be non-invasively assessed with ^123^I-mIBG myocardial SPECT imaging since the internalization of this tracer in presynaptic endings of post-ganglionic neuronal cells.^28^,^29^ Several studies proved that parameters derived by myocardial planar scintigraphy with ^123^I-mIBG play a significant prognostic role in chronic HF, with particular regard to the H/M value.^30^^–^32 However, in recent times, following the conversion from planar to SPECT tomographic techniques in clinical nuclear myocardial perfusion imaging (MPI), also ^123^I-mIBG SPECT imaging it has become progressively more common in clinical and research protocols.^5^,^33^ Early and late ^123^I-mIBG SPECT images were scored visually with 20- and 17-segment regional polar maps, also known as *bull’s-eye* maps. Additionally, segmental counts data were used to calculate regional wash-out rate (rWO) in some studies. In patients with HF, the reduced uptake of norepinephrine in presynaptic neurons determines a lower mIBG uptake (decreased H/M) and an increased WR, due to an increased release rate. Such patients are eligible for implantable cardioverter-defibrillator (ICD) implantation. The dominant observation was that the larger the extent of the ^123^I-mIBG SPECT abnormality, the higher the likelihood of ventricular tachyarrhythmia.^34^^123^I-mIBG scintigraphy was approved after the multicenter prospective study ADMIRE-HF showed a significant correlation between decreased late H/M and increased risk of HF progression, ventricular tachyarrhythmia, and death.^26^ Several studies investigated the prognostic value of ^123^I-mIBG scintigraphy in patients with chronic HF. A systematic review explored the prognostic significance of ^123^I-mIBG planar-imaging-derived parameters and indicated that patients with HF and decreased late H/M or increased WO, have a worse prognosis compared with patients with normal parameters.^27^

To date few studies, mainly evaluating relatively small samples of patients, focused on the prognostic significance of ^123^I-mIBG SPECT imaging derived parameters. Basically, data obtained by SPECT tomographic imaging have been applied in the same way as planar scintigraphic ones: to evaluate the potential correlation between ^123^I-mIBG semi-quantitative uptake scores and specific outcomes, like the occurrence of AE and SCD.^35^,^36^ Therefore, this systematic review aimed to collect sets of SPECT quantitative data from published studies to derive more accurate evidence of their prognostic value; a systematic process was adopted to avoid all possible selection bias. The methodological work-up in conducting a systematic review carries unavoidably with it some biases and limitations. First of all publication itself represents a primary source of bias as studies showing significant results are more likely to be published than studies reporting non-significant findings, therefore only complete studies taking into account the role of ^123^I-mIBG SPECT imaging in relation with planar imaging were considered. Therapy could represent another potential source of bias. In most of the studies included in this systematic review, the authors stated that the patient population was treated with either beta-blockers, Angiotensin-converting-enzyme inhibitors (ACE), or Angiotensin II Receptor Blockers (ARBs), but this was not homogeneous criteria. Only some authors specified that the patients were not undergoing treatment with tricyclic antidepressants or other medications that are known to interfere with mIBG uptake.^37^,^38^ The inclusion criteria were homogeneous for most but not all studies (NYHA class II or III, left ventricular ejection fraction LVEF ≤35%, indication for ICD implantation) hence the heterogeneity of the patient population can represent an additional source of bias. In particular, since most of the samples were extracted from patients affected by chronic HF, the absent discrimination between ischemic and non-ischemic events among such patients could be a source of error. 12 out of 16 studies assembling SPECT slices in polar maps of 17 segments were performed on patients with chronic HF, in order to predict AE or SCD in such patients. In these studies, the tracer uptake was graded on a scale of 0-4 (0=normal; 1=mildly reduced; 2=moderately reduced; 3=severely reduced; 4 = absent), and the SDS was calculated by summing the scores for each segment. All these studies stated that an increased summed score is suggestive of an increased risk for AE or SCD and associated with a worse prognosis in such patients. Most of the patients with an increased summed score also show a H/M ratio < 1.60, therefore demonstrating a good correlation between the tomographic and the planar imaging parameters. Another parameter, the regional ^123^I-mIBG rWO, calculated on SPECT imaging, was considered in one of these studies. It was calculated by subtracting the minimum rWO among 17 segments. The abnormal rWO was defined as both the rWO range and maximum rWO > mean value + 2SD obtained in 15 subjects.^39^ This SPECT parameter was significantly associated with SCD. An increased rWO, according to Doi et al.,,^40^ is associated with cardiac events in patients with chronic HF, suggesting a good prognostic value of this parameter.

Nevertheless, controversies remain on whether the best parameter to assess such risks remains the H/M on planar images. Indeed, most of the authors recognize as a major limitation to their studies the low sample size and the fact that additional studies are needed to establish the role of ^123^I-mIBG SPECT. Furthermore, the population of patients was heterogeneous since it doesn’t discriminate the etiology of chronic HF between ischemic and non-ischemic.^13^ A recent study^41^ assembling SPECT slices in polar maps of 20 instead of 17 segments was included in this review,^42^ having similar outcomes in terms of SPECT SDS in correlation with prognosis in patients with chronic HF.^43^ Several studies tried to apply ^123^I-mIBG SPECT in different ways. Somsen et al.^44^ described a new method to quantitative myocardial mIBG uptake, considering eighteen ^123^I-mIBG SPECT studies of patients with chronic HF. Myocardial uptake is calculated from the myocardial to left ventricular cavity count density ratio and the ^123^I activity in a blood sample. This was performed employing single-slice SPECT and multi-slice SPECT methods. The first compares different ROIs: mediastinum (M), right lung (L), left ventricular cavity (C), and the entire myocardium (MYO). The second analyzes semi-automatically drawn volumes of interest (VOIs). The single-slice SPECT method showed poor reproducibility than the multi-slice SPECT method that is a reproducible and accurate technique for the assessment of myocardial ^123^I-mIBG uptake but further evaluations of this method are needed.^45^^–^48 Another study^49^ examined the association between left ventricular mechanical dyssynchrony and cardiac sympathetic denervation with potential SCD events in the ADMIRE-HF (AdreView Myocardial Imaging for Risk Evaluation in Heart Failure) trial. The ADMIRE-HF subject had rest gated SPECT Technetium-99 metastable (^99m^Tc)-Tetrofosmin and ^123^I-mIBG imaging. Thanks to SPECT myocardial perfusion imaging, it was possible to determine the phase standard deviation, an index of mechanical dyssynchrony, through which it was observed an association between left ventricular mechanical dyssynchrony and SCD events in symptomatic patients with HF and reduced EF.

## Conclusions

Data from this systematic review suggest that patients affected by chronic HF, including those receiving an ICD, with a high SPECT SDS, an increased rWO, or, either way, reduced tracer uptake in specific segments of the myocardium, have an increased risk of developing AE or SCD, with a worse prognosis with respect to patients with a low SDS. In any case, it seems clear that additional studies must be performed and an automated quantitative analysis system must be adopted for ^123^I-mIBG SPECT since the lack of standardized methods makes the comparison difficult between different studies and the sharing of data between different centers, to reach the same reliable prognostic value as planar ^123^I-mIBG scintigraphy.

## Supplementary information

Below is the link to the electronic supplementary material.

(WMA 3122 kb)

(PPTX 517 kb)

(DOCX 11 kb)

## Abbreviations

HF: Heart failure
^123^I-mIBG: Iodine123-metaiodobenzylguanidine
H/M: Heart to mediastinum ratio
WR: Wash-out rate
SPECT: Single-photon emission computerized tomography
AE: Arrhythmic events
SCD: Sudden cardiac death
OS: Overall survival
MPI: Myocardial perfusion imaging
rWO: Regional wash-out rate
