# Supplementary material for: The prognostic value of 123I-mIBG SPECT cardiac imaging in heart failure patients: a systematic review
Source: J Nucl Cardiol. 2021 Jan 13;29(4):1799–809. doi: 10.1007/s12350-020-02501-w (PMC9345809; doi:10.1007/s12350-020-02501-w)
Supplement: Supplementary file 2 — (PPTX 517 kb) [file 12350_2020_2501_MOESM2_ESM.pptx]

## Slide 1
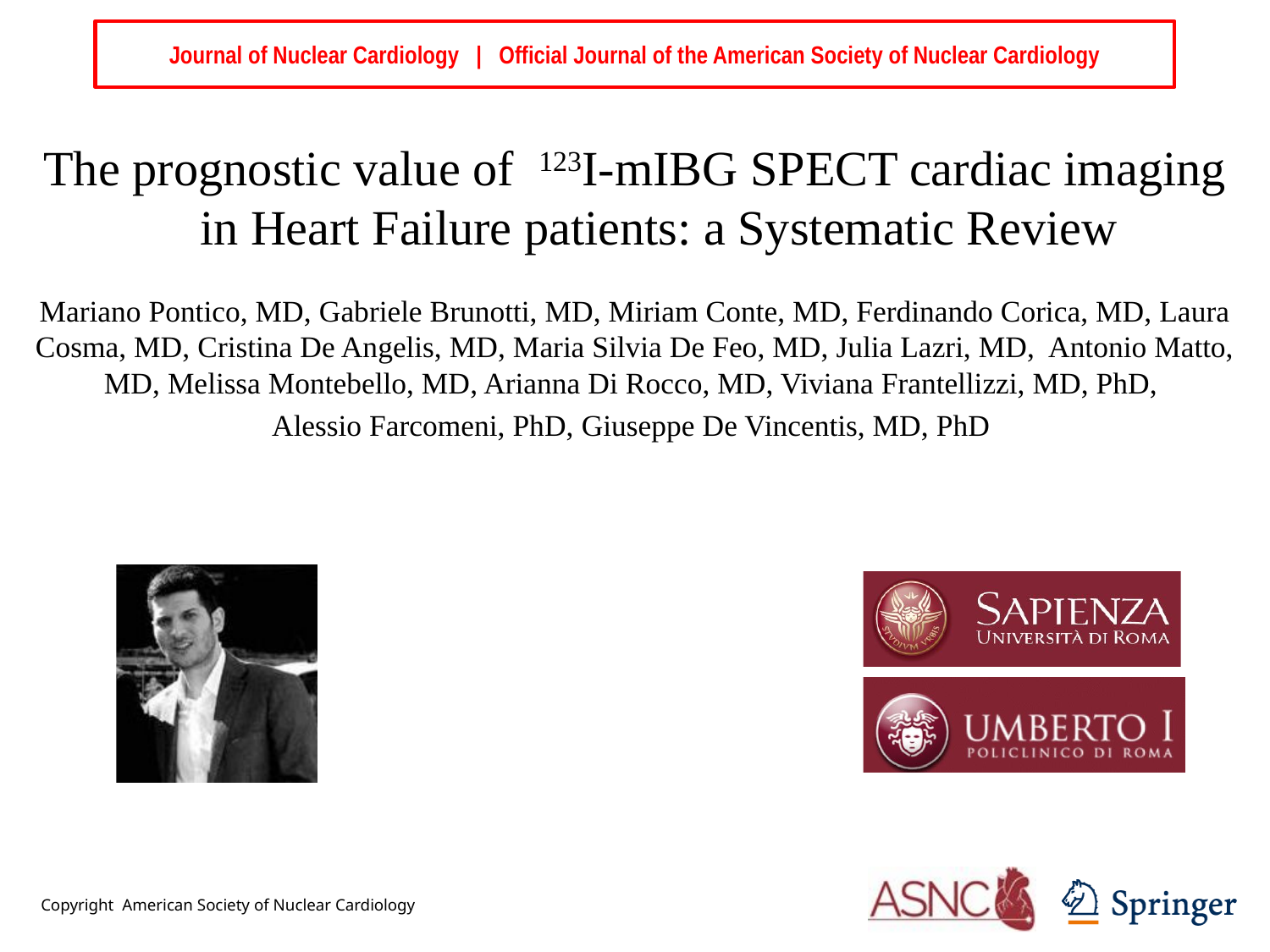

Journal of Nuclear Cardiology | Official Journal of the American Society of Nuclear Cardiology
# The prognostic value of 123I-mIBG SPECT cardiac imaging in Heart Failure patients: a Systematic Review
Mariano Pontico, MD, Gabriele Brunotti, MD, Miriam Conte, MD, Ferdinando Corica, MD, Laura Cosma, MD, Cristina De Angelis, MD, Maria Silvia De Feo, MD, Julia Lazri, MD, Antonio Matto, MD, Melissa Montebello, MD, Arianna Di Rocco, MD, Viviana Frantellizzi, MD, PhD,
Alessio Farcomeni, PhD, Giuseppe De Vincentis, MD, PhD
Copyright American Society of Nuclear Cardiology

## Slide 2
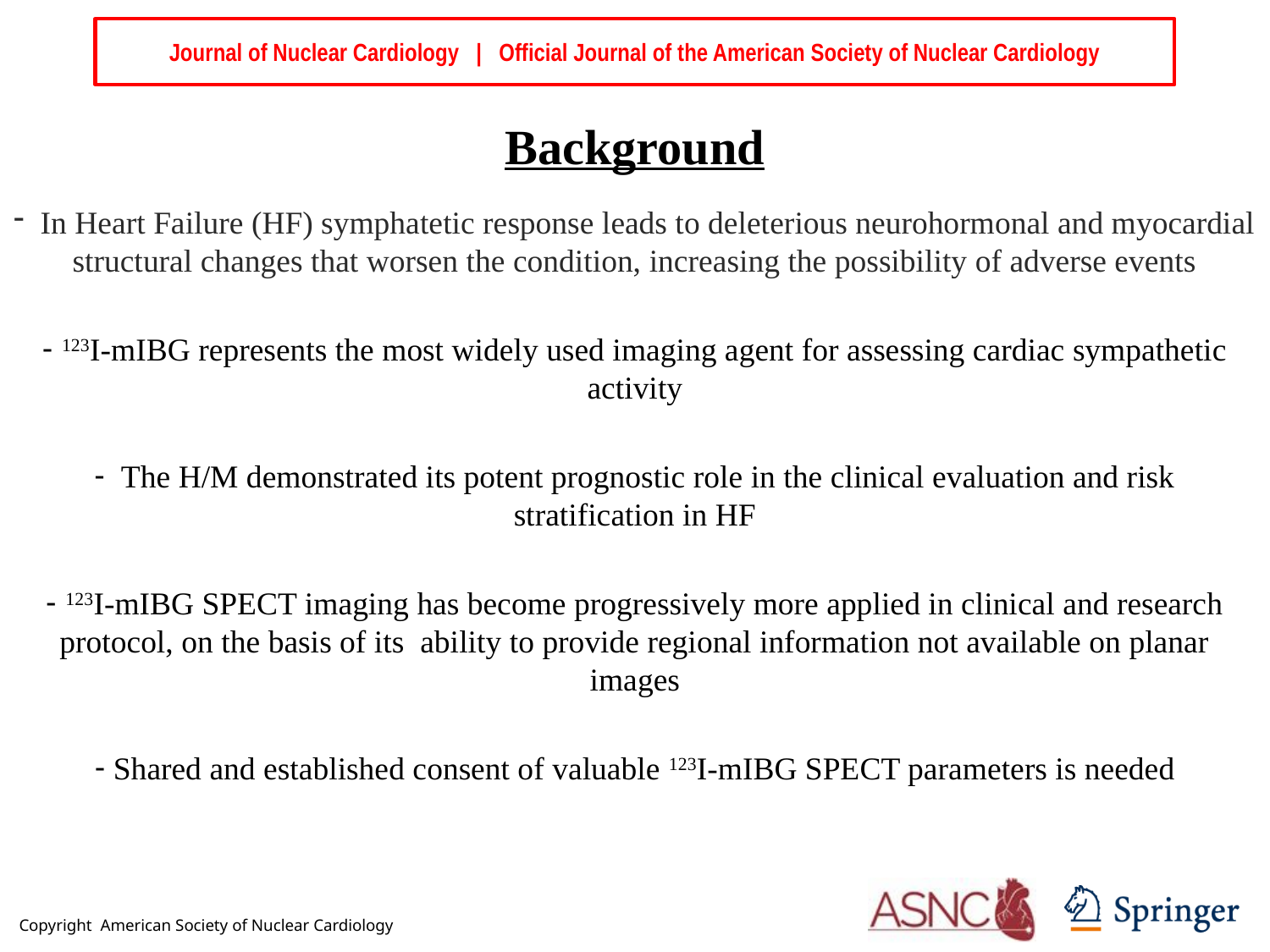

Journal of Nuclear Cardiology | Official Journal of the American Society of Nuclear Cardiology
# Background
 In Heart Failure (HF) symphatetic response leads to deleterious neurohormonal and myocardial structural changes that worsen the condition, increasing the possibility of adverse events
 123I-mIBG represents the most widely used imaging agent for assessing cardiac sympathetic activity
 The H/M demonstrated its potent prognostic role in the clinical evaluation and risk stratification in HF
 123I-mIBG SPECT imaging has become progressively more applied in clinical and research protocol, on the basis of its ability to provide regional information not available on planar images
 Shared and established consent of valuable 123I-mIBG SPECT parameters is needed
Copyright American Society of Nuclear Cardiology

## Slide 3
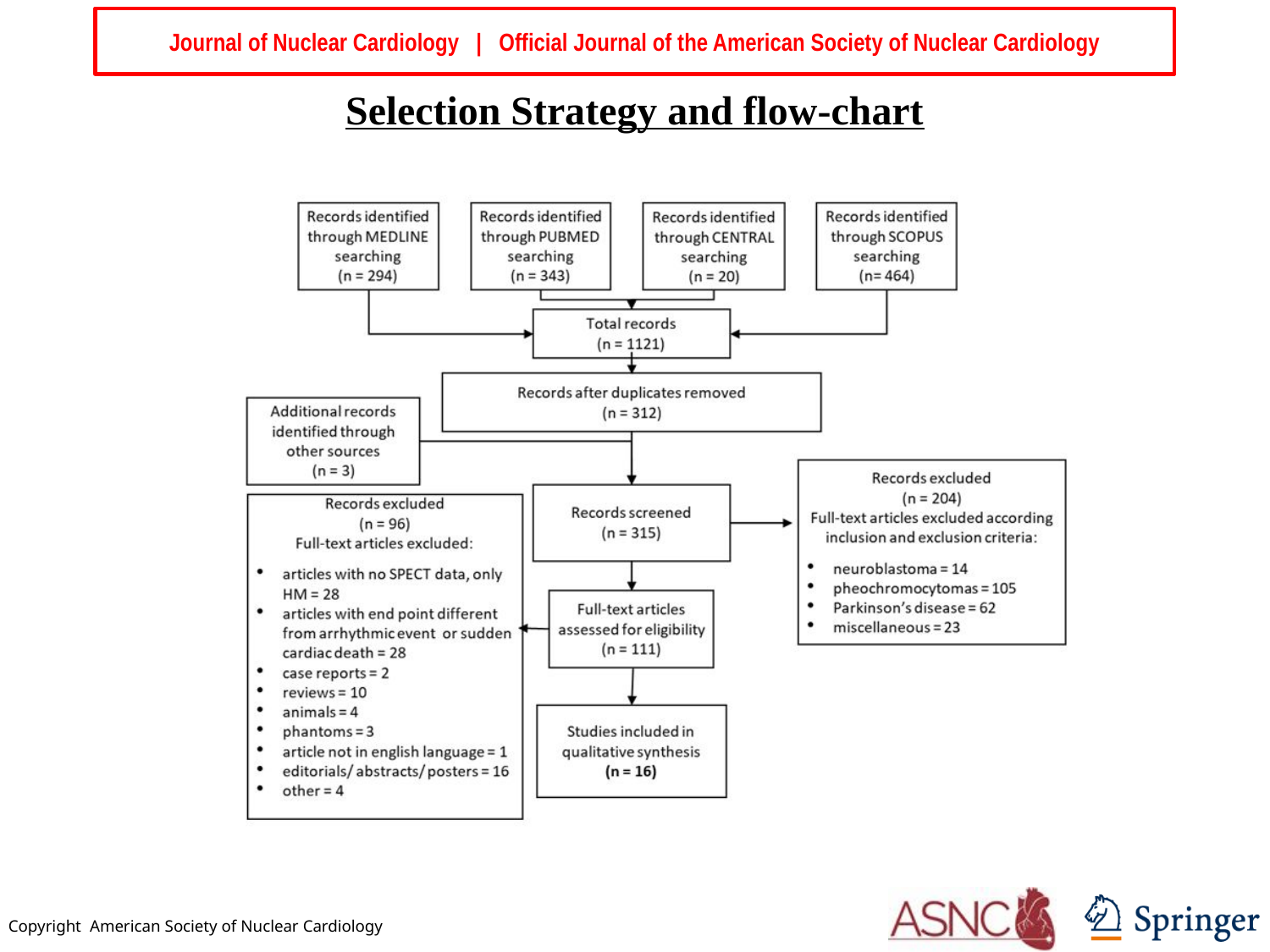

Journal of Nuclear Cardiology | Official Journal of the American Society of Nuclear Cardiology
# Selection Strategy and flow-chart
Copyright American Society of Nuclear Cardiology

## Slide 4
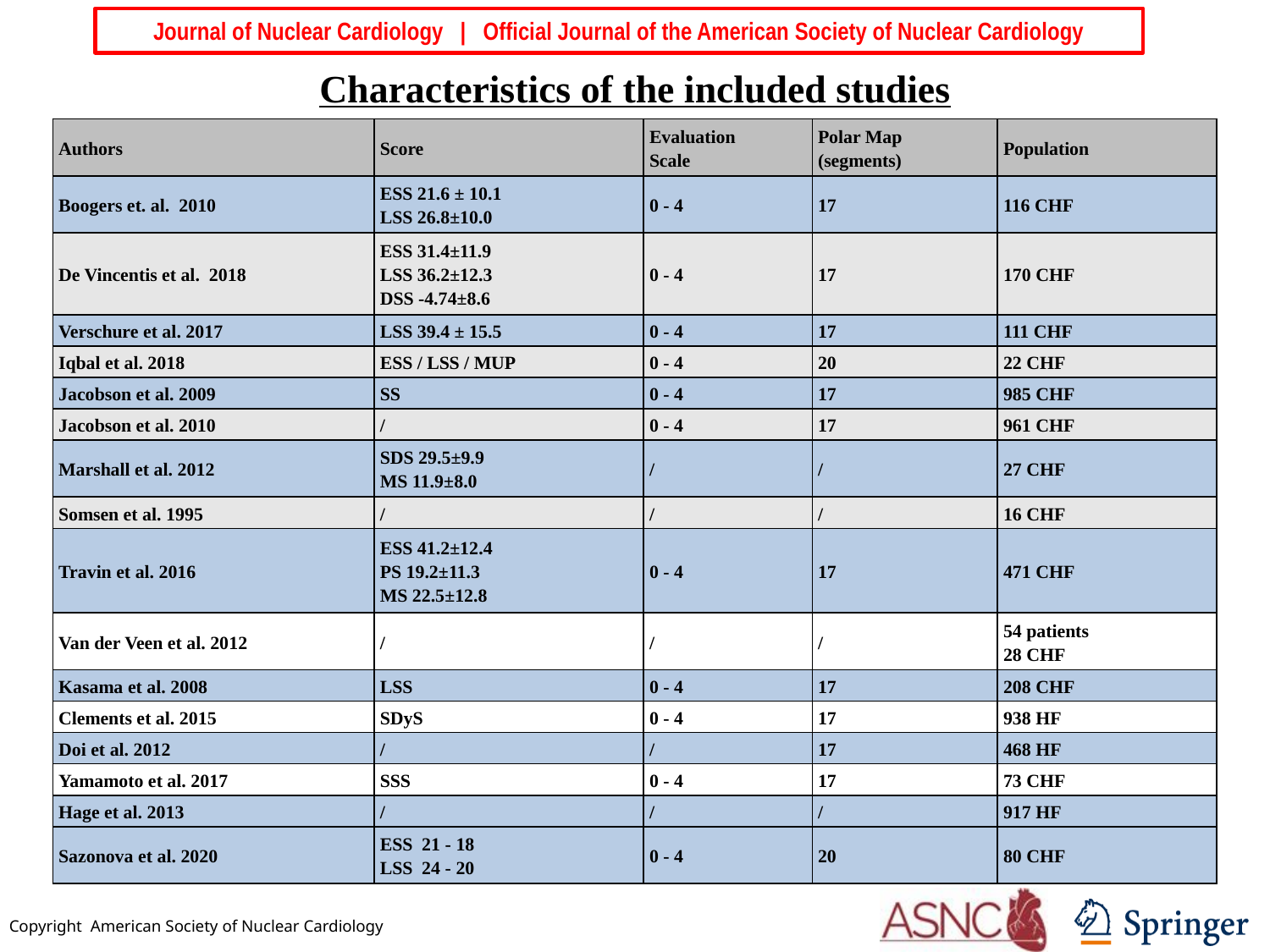

Journal of Nuclear Cardiology | Official Journal of the American Society of Nuclear Cardiology
# Characteristics of the included studies
| Authors | Score | Evaluation Scale | Polar Map (segments) | Population |
| --- | --- | --- | --- | --- |
| Boogers et. al. 2010 | ESS 21.6 ± 10.1 LSS 26.8±10.0 | 0 - 4 | 17 | 116 CHF |
| De Vincentis et al. 2018 | ESS 31.4±11.9 LSS 36.2±12.3 DSS -4.74±8.6 | 0 - 4 | 17 | 170 CHF |
| Verschure et al. 2017 | LSS 39.4 ± 15.5 | 0 - 4 | 17 | 111 CHF |
| Iqbal et al. 2018 | ESS / LSS / MUP | 0 - 4 | 20 | 22 CHF |
| Jacobson et al. 2009 | SS | 0 - 4 | 17 | 985 CHF |
| Jacobson et al. 2010 | / | 0 - 4 | 17 | 961 CHF |
| Marshall et al. 2012 | SDS 29.5±9.9 MS 11.9±8.0 | / | / | 27 CHF |
| Somsen et al. 1995 | / | / | / | 16 CHF |
| Travin et al. 2016 | ESS 41.2±12.4 PS 19.2±11.3 MS 22.5±12.8 | 0 - 4 | 17 | 471 CHF |
| Van der Veen et al. 2012 | / | / | / | 54 patients 28 CHF |
| Kasama et al. 2008 | LSS | 0 - 4 | 17 | 208 CHF |
| Clements et al. 2015 | SDyS | 0 - 4 | 17 | 938 HF |
| Doi et al. 2012 | / | / | 17 | 468 HF |
| Yamamoto et al. 2017 | SSS | 0 - 4 | 17 | 73 CHF |
| Hage et al. 2013 | / | / | / | 917 HF |
| Sazonova et al. 2020 | ESS 21 - 18 LSS 24 - 20 | 0 - 4 | 20 | 80 CHF |
Copyright American Society of Nuclear Cardiology

## Slide 5
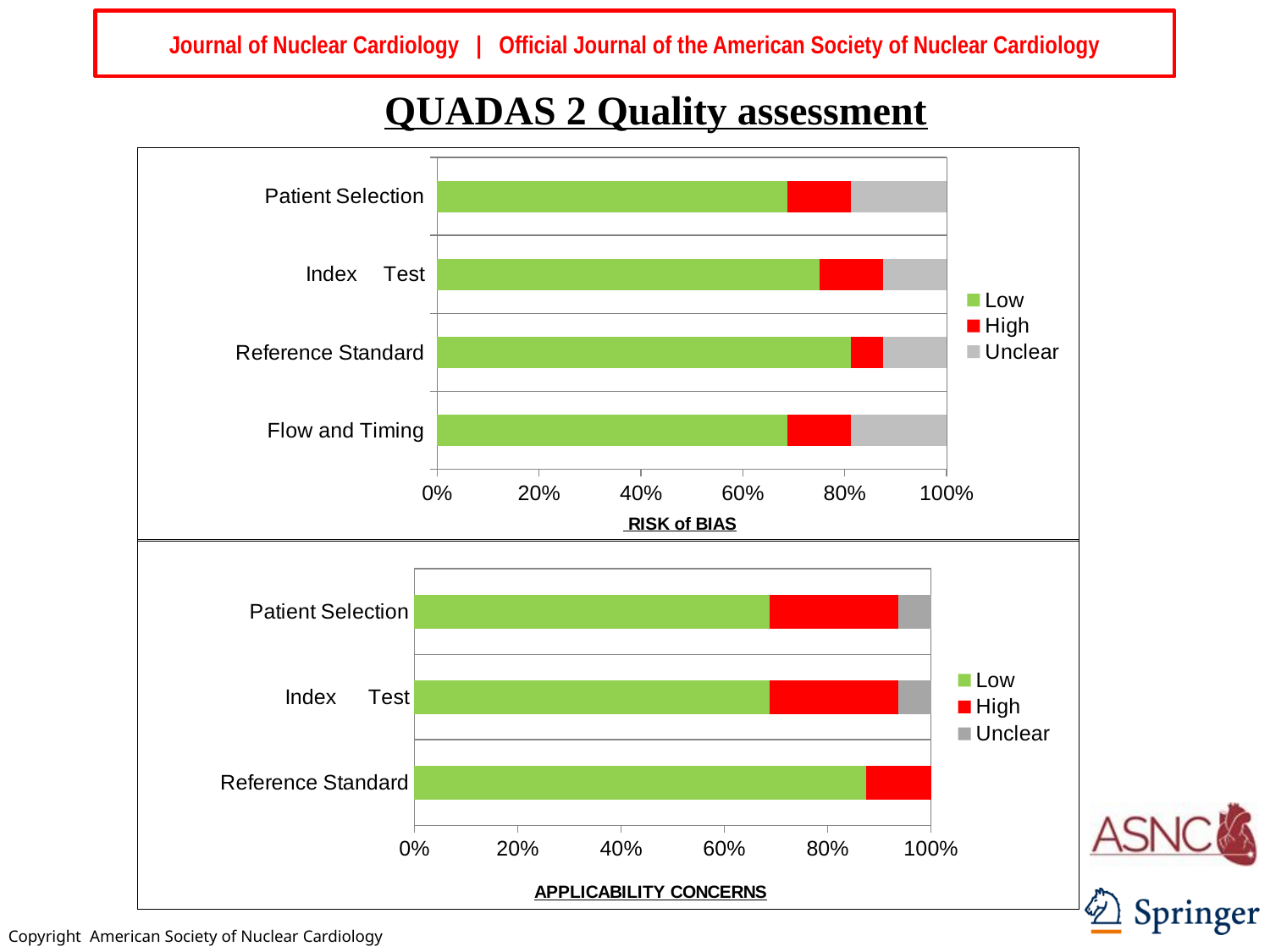

Journal of Nuclear Cardiology | Official Journal of the American Society of Nuclear Cardiology
# QUADAS 2 Quality assessment
### Chart
| Category | Low | High | Unclear |
|---|---|---|---|
| | 11.0 | 2.0 | 3.0 |
| | 12.0 | 2.0 | 2.0 |
| | 13.0 | 1.0 | 2.0 |
| | 11.0 | 2.0 | 3.0 |
### Chart
| Category | Low | High | Unclear |
|---|---|---|---|
| Patient Selection | 11.0 | 4.0 | 1.0 |
| Index Test | 11.0 | 4.0 | 1.0 |
| Reference Standard | 14.0 | 2.0 | 0.0 |
Copyright American Society of Nuclear Cardiology

## Slide 6
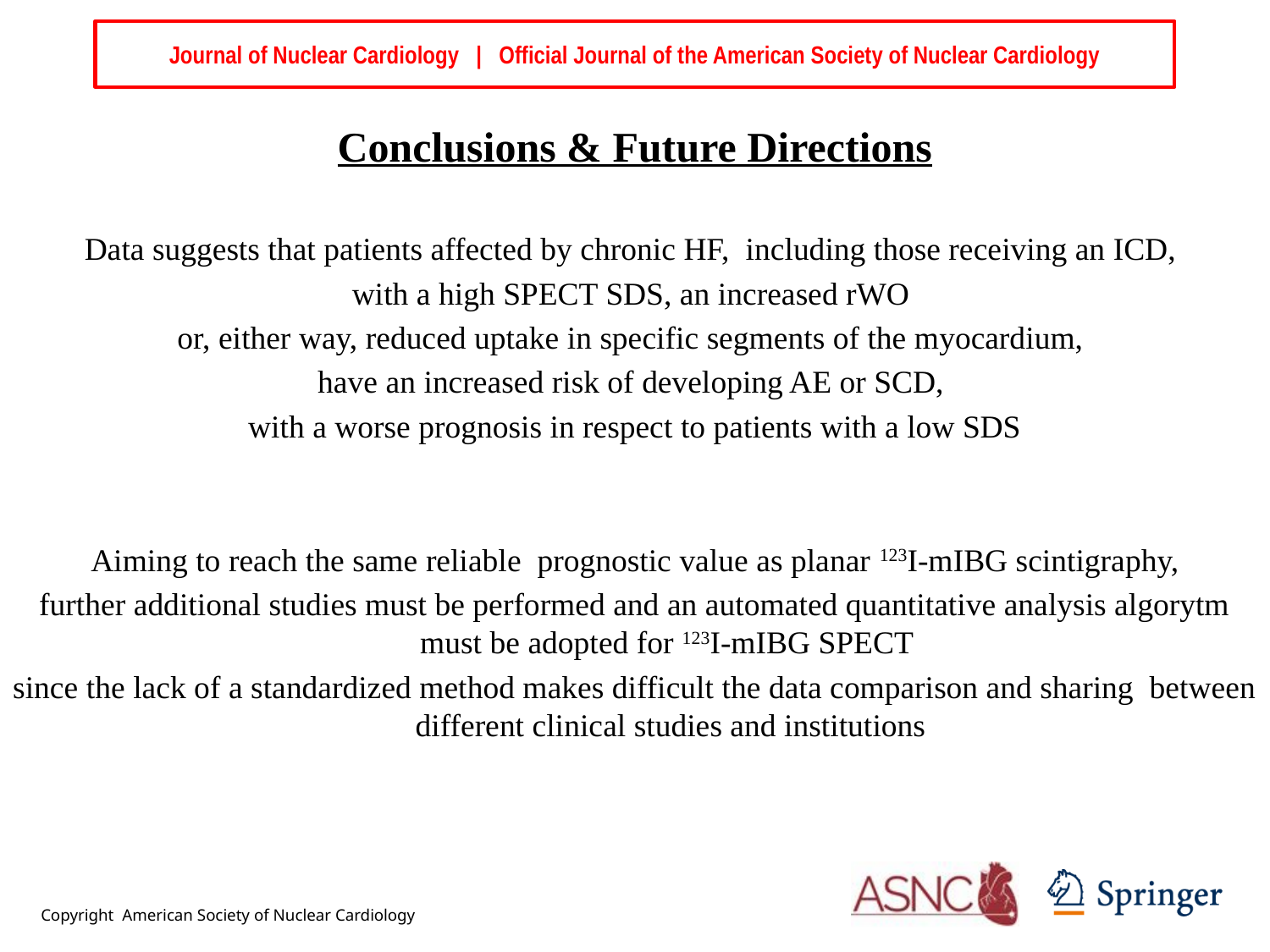

Journal of Nuclear Cardiology | Official Journal of the American Society of Nuclear Cardiology
# Conclusions & Future Directions
Data suggests that patients affected by chronic HF, including those receiving an ICD,
with a high SPECT SDS, an increased rWO
or, either way, reduced uptake in specific segments of the myocardium,
have an increased risk of developing AE or SCD,
with a worse prognosis in respect to patients with a low SDS
Aiming to reach the same reliable prognostic value as planar 123I-mIBG scintigraphy,
further additional studies must be performed and an automated quantitative analysis algorytm must be adopted for 123I-mIBG SPECT
since the lack of a standardized method makes difficult the data comparison and sharing between different clinical studies and institutions
Copyright American Society of Nuclear Cardiology
