# Supplementary material for: The prognostic value of 123I-mIBG SPECT cardiac imaging in heart failure patients: a systematic review
Source: J Nucl Cardiol. 2021 Jan 13;29(4):1799–809. doi: 10.1007/s12350-020-02501-w (PMC9345809; doi:10.1007/s12350-020-02501-w)
Supplement: Supplementary file 3 — (DOCX 11 kb) [file 12350_2020_2501_MOESM3_ESM.docx]

**Summary**

This systematic review aimed to evaluate the prognostic value of ^123^I-mIBG SPECT myocardial imaging in patients with heart failure and to assess whether semi-quantitative SPECT scores can be useful for accurate risk stratification concerning arrhythmic event and sudden cardiac death in this cohort. A systematic literature search of studies published until November 2020 regarding the application of ^123^I-mIBG SPECT in HF patients was performed, in PubMed, Scopus, Medline, Central (Cochrane Library) and Web Of Science databases. The included studies had to correlate ^123^I-mIBG SPECT scores with endpoints such as overall survival and prevention of AE and SCD in HF patients. According to the sixteen studies included, the analysis showed that 123I-mIBG SPECT scores, such as an increased SDS or rWO, as well as a reduced ^123^I-mIBG myocardial uptake, have proven to be effective in predicting AE and SCD specific risk in HF patients. Despite achieved results are promising, a more reproducible standardized method for semi-quantitative analysis and further studies with larger cohort are needed for ^123^I-mIBG SPECT myocardial imaging to be as reliable and thus accepted as the conventional ^123^I-mIBG planar myocardial imaging.
